# Lifestyle-related habits and factors before and after cardiovascular diagnosis: a case control study among 2,548 Swedish individuals

**DOI:** 10.1186/s12966-023-01446-w

**Published:** 2023-04-05

**Authors:** Amanda Lönn, Lena V. Kallings, Gunnar Andersson, Sofia Paulsson, Peter Wallin, Jane Salier Eriksson, Elin Ekblom-Bak

**Affiliations:** 1 Department of Physical Activity and Health, The Swedish School of Sport and Health Sciences, Box 5626, S-114 56 Stockholm, Sweden; 2 Women’s Health and Allied Health Professionals Theme Medical Unit Occupational Therapy and Physiotherapy, Stockholm, Sweden; 3 Department of Public Health and Caring Sciences, Family Medicine and Preventive Medicine, Uppsala University, Uppsala, Sweden; 4 Research Department, HPI Health Profile Institute, Danderyd, Sweden

**Keywords:** Physical activity, Smoking, Alcohol habits, Diet habits, Stress, Physical capacity, Lifestyle change, Cardiovascular prevention

## Abstract

**Background:**

Healthy lifestyle habits are recommended in prevention of cardiovascular disease (CVD). However, there is limited knowledge concerning the change in lifestyle-related factors from before to after a CVD event. Thus, this study aimed to explore if and how lifestyle habits and other lifestyle-related factors changed between two health assessments in individuals experiencing a CVD event between the assessments, and if changes varied between subgroups of sex, age, educational level, duration from CVD event to second assessment and type of CVD event.

**Methods:**

Among 115,504 Swedish employees with data from two assessments of occupational health screenings between 1992 and 2020, a total of 637 individuals (74% men, mean age 47 ± SD 9 years) were identified having had a CVD event (ischemic heart disease, cardiac arrythmia or stroke) between the assessments.

Cases were matched with controls without an event between assessments from the same database (ratio 1:3, matching with replacement) by sex, age, and time between assessment (*n* = 1911 controls). Lifestyle habits included smoking, active commuting, exercise, diet, alcohol intake, and were self-rated. Lifestyle-related factors included overall stress, overall health (both self-rated), physical capacity (estimated by submaximal cycling), body mass index and resting blood pressure. Differences in lifestyle habits and lifestyle-related factors between cases and controls, and changes over time, were analysed with parametric and non-parametric tests. Multiple logistic regression, OR (95% CI) was used to analyse differences in change between subgroups.

**Results:**

Cases had, in general, a higher prevalence of unhealthy lifestyle habits as well as negative life-style related factors prior to the event compared to controls. Nevertheless, cases improved their lifestyle habits and lifestyle factors to a higher degree than controls, especially their amount of active commuting (*p* = 0.025), exercise (*p* = 0.009) and non-smoking (*p* < 0.001). However, BMI and overall health deteriorated to a greater extent (*p* < 0.001) among cases, while physical capacity (*p* < 0.001) decreased in both groups.

**Conclusion:**

The results indicate that a CVD event may increase motivation to improve lifestyle habits. Nonetheless, the prevalence of unhealthy lifestyle habits was still high, emphasizing the need to improve implementation of primary and secondary CVD prevention.

**Supplementary Information:**

The online version contains supplementary material available at 10.1186/s12966-023-01446-w.

## Introduction

Regular physical activity, non-smoking, a healthy diet and modest alcohol consumption are recommended in prevention of cardiovascular disease (CVD) [1], due to their positive effects on intermediating factors [2–6] as well as direct association to the risk of CVD events and mortality [7–10]. Despite this, insufficient physical activity, overeating and daily smoking remain prevalent in the western world, with only 6% of Europe’s adult population considered having a healthy lifestyle profile [11]. Changing unhealthy lifestyle behaviours may be challenging, but several methods and behavioural change techniques used in clinical practice have been suggested and evaluated, for example, physical activity on prescription [12], multidisciplinary cardiac rehabilitation programmes [1], interactive counselling, and smartphone applications with action planning and graded task [13]. There are conflicting results if a chronical disease can motivate individuals to spontaneously adopt risk-reducing health behaviours. A Canadian prospective cohort study explored changes in lifestyle habits prior to and after being diagnosed with a disease (cancer, diabetes type II, heart disease, stroke, and respiratory disease). There was a significant decrease in smoking from before to after the event for all diagnosis except for respiratory disease. For the other lifestyle habits there where a modest change, with diabetes being associated to a positive change in physical activity and alcohol consumption and respiratory disease was associated to a positive change in alcohol consumption [14]. Hackett et al. supported the results of decrease in smoking among individuals being diagnosed with diabetes. However, they did not found any change in other lifestyle habits [15]. Meanwhile a longitudinal study, concluded that individuals being diagnosed with diabetes increased their physical activity level over time to a higher degree compared to controls [16]. In addition, cancer incidence has been shown to increase motivation for behavioural change of unhealthy lifestyle habits immediately following diagnosis [17]. However, there are to our knowledge no studies that have investigated change in lifestyle habits and related factors from before to after different CVD events using repeated assessments. The EUROASPIRE IV study concluded that a majority reported that they increased their physical activity level after a coronary event, by following specific advice from health care professionals or attending a group activity. However, this study was based on self-reports of change after the CVD event [18].

Therefore, we aimed to study if and how lifestyle habits and lifestyle-related factors changed between two health profile assessments in individuals who had a CVD event between the assessments, compared to matched controls without a CVD event between assessments. A secondary aim was to explore if any changes among cases varied between subgroups of sex, age, educational level, duration from CVD event to second assessment and type of CVD event.

## Methods

This is a nested case–control study, based on data from the Health Profile Assessment (HPA) database (www.hpi.se). A nested case–control study design use case–control methodology within an established prospective cohort and is an efficient way to investigate causal relationships [19]. HPAs have been carried out in health services in Sweden since the 1970’s and are offered to employees working for a company, or an organization connected to occupational or health related services. Participation is free of charge and optional for the individual. A HPA comprises a questionnaire including lifestyle habits and health experiences, measurements of anthropometrics and blood pressure, a submaximal cycle ergometer test, and a person-centred dialogue with a HPA coach. The Health Profile Institute is responsible for developing and standardization of the HPA, education of data collection staff, and administration of the central database.

### Participants and procedures

**Fig. 1:**
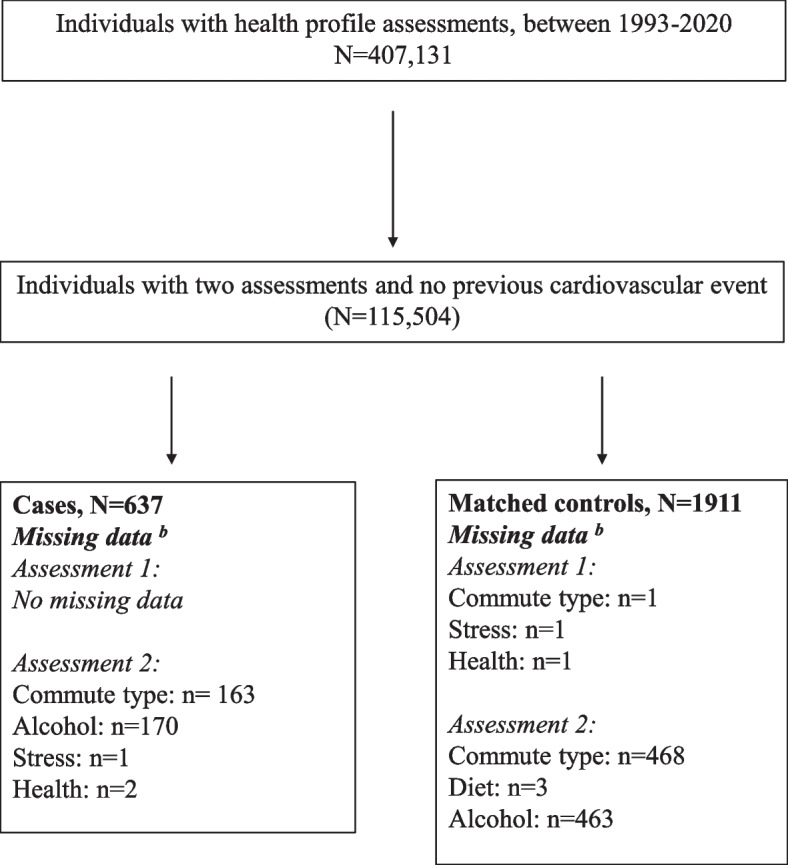
Flowchart of recruitment from the health profile assessment database. **a** two health profile assessments and no previous CVD event. **b** missing data from one assessment were replaced with data from the other assessment

In February 2021, a total of 407,131 HPAs between 1992 and 2020 were available in the database. Of those, 115,504 individuals had at least two HPAs registered in the database with no CVD event prior to the first assessment. To identify individuals with a CVD event between the two assessments, data on hospitalization due to a CVD event were retrieved from the Swedish national patient registry by linking the HPA database to the unique Swedish personal identification numbers. First time CVD events included ischemic heart disease (including heart failure, ICD 10, I20-I25, I50-I51), cardiac arrythmias (ICD 10, I46-I49) and stroke (ICD 10, I60-I66). A total of 637 (0.6%) confirmed cases with a CVD event between the two HPAs (mdn 5.92 years between assessments) were identified. These cases were matched to controls recruited from the same database, with the ratio 1:3 with permission of replacement of controls, i.e., the controls could be used repeated times. Cases were matched by sex and age at the first assessment with no tolerance of variation, and by number of days between assessments with a tolerance of variation of 180 days. Thereby, 1911 participants were eligible as controls (Fig. 1). The study was approved by the ethics board at the Stockholm Ethics Review Board (Dnr 2015/1864–31/2, 2016 9–32, 2019–05711). Informed consent was obtained from the participants prior to participation in the HPA.

### Assessment of lifestyle habits

Smoking, physically active commuting, exercise, diet, and alcohol habits were all self-rated, using a five graded pre-determined statement option (Additional file 1). To study the presence of unhealthy lifestyle habits, the variables were further dichotomized, and the following definitions were used; smoker (occasionally or daily), passive commuter (< 5 min per day physically active commuting); low exercise level (non or irregular exercise per week); poor diet habits (poor/very poor diet habits); poor alcohol habits (poor/very poor alcohol habits). The dichotomisation of smoking [8], exercise [8] and active commuting [20] are based on previous publications exploring the association to CVD events.

### Assessment of lifestyle-related factors

Overall stress and overall health were self-rated (Additional file 1). To explore often overall stress (often/very often perceived stress) and poor overall health (poor/very poor health), data were dichotomised. Physical capacity was assessed using the workload (in watts) and the rating of perceived exertion obtained while performing the submaximal Åstrand cycle test [21]. Prior to the submaximal cycle test, individuals were asked to avoid physiologic and emotional stress, smoking or using snuff one hour prior to the test, consuming a heavy meal three hours before the test and vigorous physical activity the day before the test. The participant cycled on a calibrated ergometer at an individually adapted submaximal workload (corresponding to 13–14, “Somewhat hard”, on the Borg RPE scale [22]) for 6 min. Physical capacity was expressed as submaximal workload (in watts) during the Åstrand test, divided by the Borg RPE rating minus 6 (the lowest, possible rating on the Borg scale), i.e. Watt/Borg RPE-6. This procedure, rather than the normal calculation of estimated maximal oxygen consumption from the Åstrand test, was used to avoid direct use of heart rate response which is affected by medication of betablockers. Body mass was assessed with a calibrated scale to the nearest 0.5 kg, with the individual wearing light-weight clothing. Height was assessed using a wall-mounted stadiometer. Body mass index (BMI) was calculated from the individual’s body weight and height, (kg/m^2^). Systolic and diastolic blood pressure were measured manually in the right arm using the standard auscultatory method after 20 min of seated resting. Highest educational attainment at the time of each HPA was derived from the Swedish education nomenclature 2000-registry (S*tatistics Sweden*) as years of education.

### Statistics

Lifestyle-related habits and factors at baseline and follow-up, and change over time of these, were presented as continuous as well as dichotomized variables. Continuous variables were checked for normal distribution and presented as mean (SD) or median (q1 and q3). Lifestyle habits and perceived overall stress and health (non-parametric data) were presented as frequency and relative frequency. For skewed data, the Mann Whitney U-test was used to analyse differences between cases and controls, and the Wilcoxon signed-rank test to analyse differences over time. For normally distributed data, differences between groups and over time were analysed by unpaired and paired-sample t test, respectively. To explore the clustering of unhealthy lifestyle habits and overall stress, an index was created. Each unhealthy lifestyle-related habit (low exercise level, passive commuting, poor diet habits, smoking, poor alcohol habits) and unhealthy lifestyle-related factor (often overall stress) was appointed one point, resulting in a possible range of 0 to 6 with 0 being considered as non-unhealthy lifestyle habits. To compare differences in proportion to every separate unhealthy lifestyle habit between cases and controls, the Chi2 test was used. McNemars test was used to identify changes in percentages of each unhealthy lifestyle habit over time. Multiple logistic regression analyses were used to calculate adjusted odds ratios (ORs) with 95% confidence intervals (Cis) for positive and negative change in unhealthy lifestyle habits among the following demographics – sex, age, educational level, time between CVD event and second assessment, and type of CVD event. Internal dropouts were handled by missing data from one assessment being replaced with data from the other assessment, i.e., presuming no change. The level of difference was set to *p* < 0.05 and all the above analyses were performed using IBM SPSS (V.27.0.0.1).

## Results

### Participants

A total of 637 cases (40% ischemic heart disease, 38.5% cardiac arrythmias, 21.5% stroke) and 1,911 matched controls were included. The mean age was 47 years (SD 9 years), 74% were men, with no difference in educational level (education ≤ 12 years; 82% versus 80%) between cases and controls.

### Lifestyle habits and related factors in cases vs controls

Prior to the CVD event, cases reported less active commuting (*p* = 0.011), more smoking (*p* = 0.002), higher overall stress (*p* = 0.038), and poorer overall health (*p* = 0.032) compared to controls. In addition, cases had higher BMI (*p* = 0.026) and diastolic blood pressure (*p* = 0.01) (Table 1).

**Table 1:** Lifestyle habits and lifestyle-related factors in cases and controls at baseline and second assessment

	**Cases**			**Controls**			**Cases vs. Controls**		
	Baseline	2nd assessment	Change within cases	Baseline	2nd assessment	Change within controls	Baseline	2nd assessment	Change over time
**Smoking, n (%), *n* = 2548**			*p* < 0.001			*p* < 0.001	*p* = 0.002	*p* = 0.36	*p* < 0.001
≥ 20 cig/day	5 (1%)	1 (0%)		17 (1%)	11 (1%)				
11–19 cig/day	46 (7%)	12 (2%)		52 (3%)	64 (3%)				
1–10 cig/day	42 (7%)	34 (5%)		116 (6%)	95 (5%)				
Occasionally	50 (8%)	27 (4%)		151 (8%)	75 (4%)				
Never	494 (78%)	563 (88%)		1575 (82%)	1666 (87%)				
**Active commuting, n (%), *n* = 2519**			< 0.001			*p* < 0.001	*p* = 0.011	*p* = 0.81	*p* = 0.007
< 5 min/day	437 (69%)	275 (59%)		1210 (64%)	845 (58%)				
5–9 min/day	55 (9%)	39 (8%)		170 (9%)	124 (9%)				
10–19 min/day	60 (10%)	56 (12%)		216 (11%)	200 (14%)				
20–29 min/day	49 (8%)	46 (10%)		154 (8%)	131 (9%)				
At least 30 min/day	30 (5%)	52 (11%)		137 (7%)	160 (11%)				
**Exercise, n (%), *n* = 2548**			< 0.001			*p* < 0.001	*p* = 0.798	*p* = 0.015	*p* = 0.009
Never	65 (10%)	55 (9%)		226 (12%)	169 (9%)				
Irregular	172 (27%)	117 (18%)		496 (26%)	475 (25%)				
1–2 times per week	223 (35%)	216 (34%)		661 (35%)	589 (31%)				
3–5 times per week	163 (26%)	215 (34%)		458 (24%)	586 (31%)				
At least 6 times per week	14 (2%)	34 (5%)		70 (4%)	92 (5%)				
**Diet habits, n (%), *n* = 2548**			< 0.001			*p* < 0.001	*p* = 0.398	*p* = 0.62	*p* = 0.23
Very poor	10 (2%)	1 (0%)		34 (2%)	4 (0%)				
Poor	50 (8%)	18 (3%)		165 (9%)	46 (2%)				
Neither poor nor good	238 (37%)	146 (23%)		652 (34%)	475 (25%)				
Good	302 (47%)	395(62%)		927 (49%)	1145 (60%)				
Very good	37 (6%)	77 (12%)		133 (7%)	238 (13%)				
**Alcohol habits, n (%), *n* = 2518**			< 0.001			*p* < 0.001	*p* = 0.690	*p* = 0.020	*p* = 0.015
Very poor	0 (0%)	0 (0%)		6 (0%)	0 (0%)				
Poor	32 (5%)	12 (3%)		87 (5%)	68 (5%)				
Neither poor nor good	353 (56%)	181 (39%)		1065 (57%)	606 (43%)				
Good	182 (29%)	168 (36%)		563 (30%)	513 (36%)				
Very good	65 (10%)	101 (22%)		164 (9%)	237 (17%)				
**Overall stress, n (%), *n* = 2548**			0.011			*p* < 0.001	*p* = 0.038	*p* = 0.04	*p* = 0.87
Very often	12 (2%)	13 (2%)		31 (2%)	23 (1%)				
Often	68 (11%)	54 (9%)		168 (9%)	128 (7%)				
Now and then	242 (38%)	220 (35%)		688 (36%)	640 (36%)				
Occasionally	259 (41%)	274 (43%)		824 (43%)	870 (46%)				
Never	56 (9%)	75 (12%)		199 (10%)	250 (13%)				
**Overall health, n (%), *n* = 2548**			0.006			*p* = 0.039	*p* = 0.032	*p* < 0.001	*p* < 0.001
Very poor	3 (1%)	2 (0%)		8 (0%)	5 (0%)				
Poor	34 (5%)	46 (7%)		68 (4%)	75 (4%)				
Neither poor nor good	173 (27%)	191 (30%)		493 (26%)	447 (23%)				
Good	364 (57%)	346 (55%)		1102 (58%)	1120 (59%)				
Very good	63 (10%)	50 (8%)		239 (13%)	264 (14%)				
***Physical capacity***^a^***, median (Q1-Q3), n = 2548***	16 (13 to 19)	15 (13 to 18)	< 0.001	17 (14 to 20)	15 (13 to 18)	*p* < 0.001	*p* = 0.061	*p* = 0.08	*p* = 0.98
***BMI, kg/m2, median (Q1-Q3), n* = *2546***	26 (24 to 28)	26 (24 to 29)	< 0.001	25 (23 to 28)	26 (24 to 28)	*p* < 0.001	*p* = 0.026	*p* = 0.01	*p* < 0.001
***Systolic blood pressure, mmHg, mean (SD), n* = *2535***	130 (16)	129 (14)	0.60	127 (15)	130 (16)	*p* < 0.001	*p* = 0.20	*p* = 0.20	*p* = 0.009
***Diastolic blood pressure, mmHg, mean (SD), n* = *2539***	80 (11)	80 (10)	0.58	79 (10)	81 (10)	*p* < 0.001	*p* = 0.01	*p* = 0.05	*p* = 0.15

^a^watt/Borg RPE-6

In general, both cases and controls improved lifestyle habits and related factors by the second assessment. However, cases increased active commuting (*p* = 0.007), exercise (*p* = 0.009), decreased smoking (*p* < 0.001) and improved alcohol habits (*p* = 0.015), to a greater extent than controls. On the contrary, cases reported poorer overall health at the second assessment (*p* < 0.001). Physical capacity was lower in both cases and controls at the second assessment (*p* < 0.001), with no differences between the groups (*p* = 0.980). BMI increased more in cases than in controls (*p* < 0.001), while systolic and diastolic blood pressure increased in controls (*p* < 0.001), but not cases.

### Unhealthy lifestyle habits and perceived stress and health among cases and controls

At the first assessment, the most prevalent unhealthy lifestyle habits in both cases and controls were passive commuting (70% and 64%) and low exercise levels (38% and 37%), followed by smoking (22% and 18%) (Table 2). Between the two assessments, the proportion of every unhealthy lifestyle habit had decreased among cases (*p* < 0.001) except for overall stress. Compared to controls, a significantly larger proportion (*p* < 0.05) of cases transferred to non-smokers (10% vs 5%), active commuters (10% versus 4%) and doing at least two weekly exercise sessions (10% versus 4%).

**Table 2:** Prevalence in unhealthy lifestyle habits, perceived stress and health among cases and controls

	**Cases**			**Controls**			**Cases vs. Controls**
	**Baseline**	**2nd assessment**	**Change**	**Baseline**	**2nd assessment**	**Change**	**Change**
**Smoker**^a^	22% (19% to 26%)	12% (9% to 14%)	< 0.001	18% (16% to 19%)	13% (11% to 14%)	*p* < 0.001	*p* < 0.001
**Passive commuter**^b,d^	70% (66% to 73%)	60% (56% to 64%)	< 0.001	64% (62% to 66%)	60% (57% to 62%)	*p* < 0.001	*p* = 0.025
**Low exercise level**^c^	37% (33% to 41%)	27% (24% to 31%)	< 0.001	38% (36% to 40%)	34% (32% to 36%)	*p* < 0.001	*p* = 0.009
**Poor diet habits**	9% (7% to 12%)	3% (2% to 5%)	< 0.001	10% (9% to 12%)	3% (2% to 4%)	*p* < 0.001	*p* = 0.40
**Poor alcohol habits**^e^	5% (3% to 7%)	3% (2% to 5%)	< 0.001	5% (4% to 6%)	5% (4% to 6%)	*p* < 0.001	*p* = 0.10
**Often overall stress**	13% (10% to 15%)	11% (8% to 13%)	0.28	11% (9% to 12%)	8% (7% to 9%)	*p* = 0.002	*p* = 0.73
**Poor overall health**	6% (4% to 8%)	7% (6% to 10%)	0.19	4% (3% to 5%)	4% (3% to 5%)	*p* = 0.78	*p* = 0.20

Data presented as % (95% CI)

^a^occasionally or daily

^b^ < 5 min per day physically active commuting

^c^irregular or non per week

^d^cases *n* = 631, controls *n* = 1888

^e^cases *n* = 632, controls *n* = 1886

**Fig. 2:**
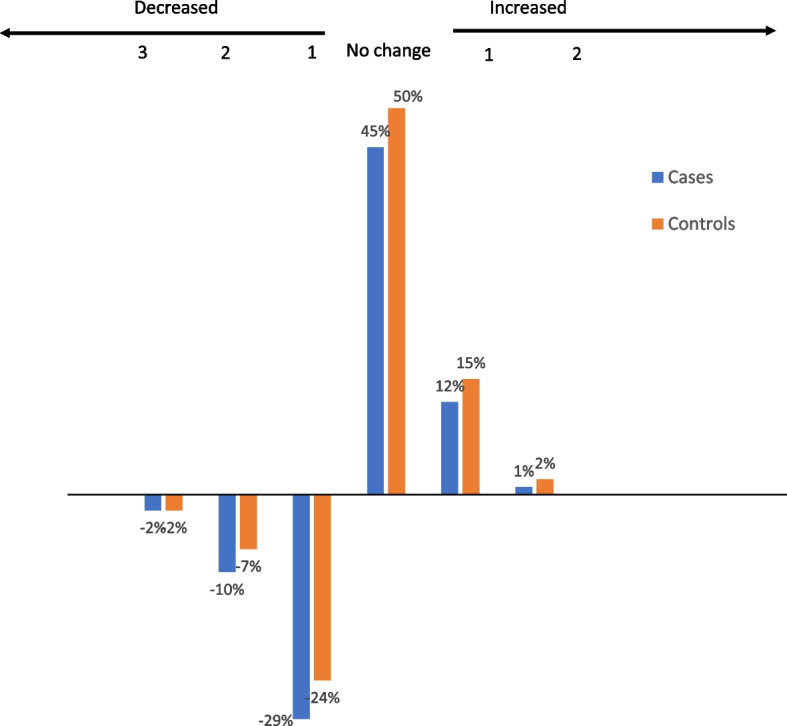
Change in prevalence of unhealthy lifestyle habits and stress among cases and controls between assessments

In analyses of the index, the mean total number of unhealthy lifestyle habits and stress were higher in cases compared to controls at the first assessment prior to the CVD event, 1.59 (SD 1.05) vs. 1.45 (SD 1.02) (*p* = 0.029). However, the mean number decreased to a higher degree among cases, -0.41 (SD 0.96) compared to controls -0.24 (SD 0.95), (*p* < 0.01), (Fig. 2).

**Fig. 3:**
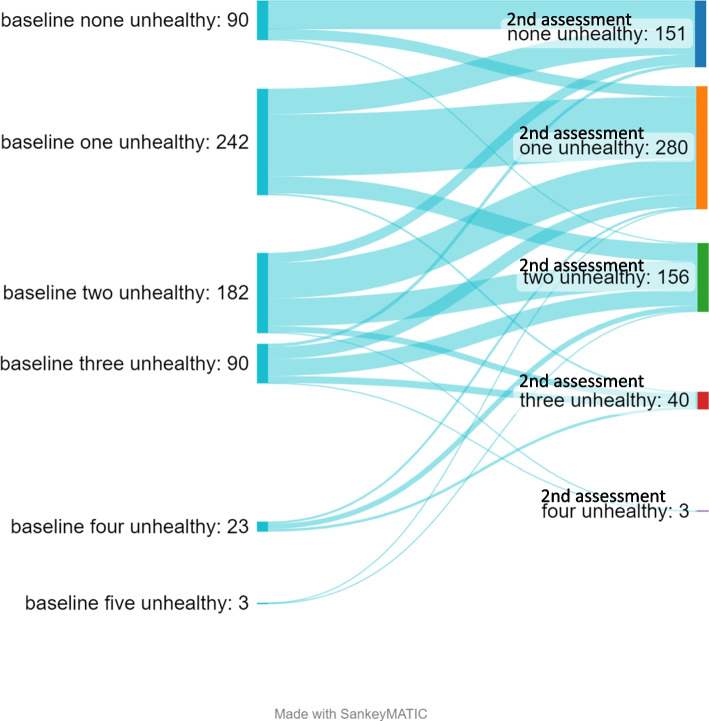
Individual differences in change in number of unhealthy lifestyle habits among cases

There were individual differences in change in number of unhealthy lifestyle habits (Fig. 3). However, most individuals did no change or changed one unhealthy lifestyle habit to follow up. All cases with four or five unhealthy lifestyle habits at baseline improved their number of unhealthy lifestyle habits at follow-up.

### Changes among cases in relation to sub-groups

Changes in lifestyle habits, perceived stress, and health among cases between assessments in relation to sex, age, educational level, duration from the CVD event to the second assessment, and type of CVD are presented in Table 3. In general, there were small differences in change between subgroups, however younger individuals were less prone to make positive change of exercise OR 0.51 (95% CI 0.35–0.76) and diet OR 0.20 (95% CI 0.10–0.39) and women were less prone to make a positive change in stress OR 0.47 (95% 0.26–0.86) compared to men. For the index, women were less prone to make a positive change compared to men, OR 0.65 (95% CI 0.44–0.95), while individuals with ischemic heart disease were more prone to make a positive change of the index compared to individuals with cardiac arrythmias, OR 1.86 (95% CI 1.28–2.70). Differences in the proportion of the unhealthy lifestyle habits in subgroups at baseline and second assessment are described in Additional file 2.

**Table 3:** Odds ratio (95% CI) for positive (left) and negative (right) change in unhealthy lifestyle habits, stress and health among cases in relation to sub-groups

	**Positive change OR (95% CI)**	**Negative change OR (95% CI)**
**Smoking (*n* = 637)**		
Women vs men	0.63 (0.37–1.07)	4.39 (0.53–36.65)
< 47 years vs ≥ 47 years	1.03 (0.63–1.69)	0.32 (0.08–1.28)
Secondary school vs college	0.64 (0.33–1.30)	0.52 (0.06–4.19)
≤ 1 year CVD event vs > 1 year CVD event^a^	1.21 (0.70–2.11)	1.61 (0.33–7.77)
Ischemic heart disease vs cardiac arrhythmia	*2.39 (1.31–4.35)*	0.78 (0.17–3.60)
Stroke vs cardiac arrhythmia	*2.19 (1.12–4.28)*	1.70 (0.36–7.65)
**Passive commuting (*n* = 631)**		
Women vs men	0.71 (0.42–1.20)	0.65 (0.27–1.59)
< 47 years vs ≥ 47 years	0.83 (0.52–1.33)	1.14 (0.51–2.59)
Secondary school vs college	0.71 (0.37–1.36)	0.36 (0.08–1.56)
≤ 1 year CVD event vs > 1 year CVD event ^a^	1.43 (0.82–2.49)	1.69 (0.62–4.56)
Ischemic heart disease vs cardiac arrhythmia	1.18 (0.69–2.00)	2.07 (0.64–6.70)
Stroke vs cardiac arrhythmia	0.91 (0.48–1.73)	1.46 (0.43–4.90)
**Low exercise level (*n* = 637)**		
Women vs men	0.92 (0.59–1.45)	0.91 (0.50–1.67)
< 47 years vs ≥ 47 years	*0.51 (0.35–0.76)*	1.36 (0.79–2.34)
Secondary school vs college	1.26 (0.78–2.04)	0.72 (0.34–1.52)
≤ 1 year CVD event vs > 1 year CVD event ^a^	1.03 (0.67–1.60)	0.97 (0.54–1.74)
Ischemic heart disease vs cardiac arrhythmia	*1.67 (1.06–2.62)*	*0.51 (0.28–0.93)*
Stroke vs cardiac arrhythmia	1.49 (0.88–2.53)	0.61 (0.30–1.23)
**Poor diet habits (*n* = 637)**		
Women vs men	*3.94 (1.58–9.85)*	1.01 (0.25–4.01)
< 47 years vs ≥ 47 years	*0.20 (0.10–0.39)*	0.31 (0.08–1.20)
Secondary school vs college	*0.34 (0.12–0.97)*	0.37 (0.05–2.97)
≤ 1 year CVD event vs > 1 year CVD event^a^	1.15 (0.58–2.24)	0.41 (0.12–1.39)
Ischemic heart disease vs cardiac arrhythmia	1.81 (0.88–3.74)	0.52 (0.12–2.17)
Stroke vs cardiac arrhythmia	*2.94 (1.33–6.48)*	0.61 (0.12–2.12)
**Poor alcohol habits (*n* = 632)**		
Women vs men	1.13 (0.39–3.29)	na (-)
< 47 years vs ≥ 47 years	2.39 (0.88–6.48)	1.89 (0.36–9.96)
Secondary school vs college	1.81 (0.63–5.26)	0.81 (0.10–6.85)
≤ 1 year CVD event vs > 1 year CVD event^a^	0.56 (0.22–1.42)	1.05 (0.20–5.47)
Ischemic heart disease vs cardiac arrhythmia	0.37 (0.11–1.25)	0.76 (0.15–3.84)
Stroke vs cardiac arrhythmia	1.55 (0.55–4.39)	0.69 (0.07–6.79)
**Often overall stress (*n* = 637)**		
Women vs men	*0.47 (0.26–0.86)*	0.73 (0.36–1.50)
< 47 years vs ≥ 47 years	0.61 (0.35–1.08)	*0.51 (0.27–0.98)*
Secondary school vs college	1.23 (0.63–2.40)	*2.82 (1.47–5.40)*
≤ 1 year CVD event vs > 1 year CVD event ^a^	1.43 (0.73–2.78)	0.55 (0.29–1.04)
Ischemic heart disease vs cardiac arrhythmia	1.65 (0.88–3.10)	1.65 (0.84–3.23)
Stroke vs cardiac arrhythmia	0.88 (0.40–1.93)	0.41 (0.13–1.26)
**Poor overall health (*n* = 637)**		
Women vs men	1.40 (0.50–3.91)	0.66 (0.31–1.43)
< 47 years vs ≥ 47 years	0.73 (0.32–1.70)	0.64 (0.32–1.31)
Secondary school vs college	0.37 (0.09–1.61)	1.57 (0.71–3.49)
≤ 1 year CVD event vs > 1 year CVD event^a^	0.75 (0.31–1.8)	0.88 (0.41–1.90)
Ischemic heart disease vs cardiac arrhythmia	0.39 (0.14–1.04)	1.85 (0.80–4.24)
Stroke vs cardiac arrhythmia	0.51 (0.16–1.59)	1.65 (0.64–4.22)
**Total index**^b^** (*n* = 630)**		
Women vs men	*0.65 (0.44–0.95)*	1.44 (0.80–2.60)
< 47 years vs ≥ 47 years	0.73 (0.52–1.01)	0.95 (0.60–1.52)
Secondary school vs college	0.79 (0.52–1.21)	0.91 (0.50–1.66)
≤ 1 year CVD event vs > 1 year CVD event^a^	1.28 (0.88–1.84)	0.84 (0.51–1.38)
Ischemic heart disease vs cardiac arrhythmia	*1.86 (1.28–2.70)*	0.88 (0.53–1.44)
Stroke vs cardiac arrhythmia	1.51 (0.97–2.34)	0.44 (0.21–0.92)

^a^time from CVD event to second assessment

^b^Total index of unhealthy lifestyel habits and often overall stress

## Discussion

The main findings in this nested case–control study are 1) that individuals experiencing a CVD event in between two health assessments had a higher prevalence of unhealthy lifestyle habits and lifestyle-related factors compared to controls prior to the event, 2) cases made improvements to a higher degree than controls, especially the amount of active commuting, exercise and smoking, 3) BMI and overall health declined to a greater extent among cases compared to controls between assessments, with a decline in physical capacity for both cases and controls. Overall, experiencing a CVD event may contribute to higher motivation to improve lifestyle habits, with small differences between demographic subgroups.

Studies focusing on the effect of a CVD event on lifestyle change are, to our knowledge, limited. One qualitative study among myocardial infarction survivors emphasised that the event was a major motivation to improve their lifestyle habits. The study did not, however, explore if there was an actual change [23]. The significant improvements for all lifestyle habits in the present study differs from a study including individuals with different diagnosis, only finding a significant decrease in smoking for individuals with heart disease and stroke. Although, when exploring the clustering of unhealthy lifestyle habits our results were similar to the findings in cancer survivors, which did also improve risk behaviours in relation to experiencing a life-threating event [17].

The significantly larger improvement in cases may also be partly explained by regression towards the mean, with cases having a higher prevalence of unhealthy lifestyle habits before the event compared to controls. The same pattern was seen among individuals with ischemic heart disease or stroke, where larger improvements were made compared to individuals with cardiac arrythmias who had better lifestyle habits prior to the event. Thereto, differences between CVD groups could be due to a more established prevention support for individuals experiencing a stroke or ischemic heart disease [1]. Emphasising the need for improved support for individuals with cardiac arrhythmias in clinical practice. Other groups with lower odds for making a positive change and thereby should be a prioritised group for prevention interventions was younger individuals (exercise and diet) and women (stress and unhealthy lifestyle habit index). Interestingly, the controls also improved their lifestyle habits over time. Perhaps the general improvement in lifestyle habits among both cases and controls could potentially be attributed to the person-centred dialogue with a HPA coach.

Although a significant number of cases in the present study improved their lifestyle habits, a large proportion still had unhealthy lifestyle habits, with 12% being smokers, 60% being passive commuters and 27% having an insufficient level of exercise after their CVD event. This is in line with other studies assessing lifestyle habits at one timepoint after a CVD event, that conclude that approximately 12–25% were smokers [8, 18, 24, 25] and 33–66% were considered having an insufficient level of physical activity at moderate and vigorous intensity [8, 18, 25]. The high prevalence of unhealthy lifestyle habits after a CVD event may be a sign of unsuccessful implementation of prevention interventions. This is in line with results from a study among health care professionals at in- and outpatient cardiac care where the authors concluded that only a low amount of support was given to patients to improve lifestyle habits. This was despite the health care professionals considering it important to work with lifestyle habits and expressing a wish to improve this support [26].

The need of increased support was also evident among individuals surviving a myocardial infarction. Patients reported a perceived feeling of being burdened with the responsibility of changing lifestyle habits [23]. Altogether, this highlights that the window of opportunity, when patients have an increased level of motivation, is not made full use of by the health care sector. Using this opportunity would improve lifestyle habits among patients after a CVD event, furthering the recommendations of the international guidelines of prevention.

## Strengths and limitations

We used a large cohort of Swedish men and women to identify cases and controls in this nested case–control study. A major strength was the study design with cases and controls being age, gender and duration between assessments matched from the same population (national HPI database). Thereto, the inclusion of standardized, repeated assessments of lifestyle-related factors at two timepoints as opposed to retrospective reporting, which can minimize recall bias. Although exercise and commuting habits were self-reported [27], the use of questions with predetermined answer categories in the present study have been reported to provide superior validity for physical activity levels compared to open answer options [28]. The dichotomisation of healthy vs unhealthy lifestyle habits can contribute to information loss, however it describes the change between healthy and unhealthy lifestyle habits over time, not a change in already healthy lifestyle habits, which is of clinical relevance. The dichotomisation was constructed on subjective decisions, although based on results from previous publications [8, 20].

Another limitation was that we used a non-validated assessment of physical capacity. However, estimation of cardiorespiratory fitness using heart rate response during the submaximal test would have induced large errors among the participants with beta-receptor block treatment. Previous studies conclude that cardiorespiratory fitness decreases with age [29], which might have been the cause of the decrease in physical capacity at the second assessment. Conclusions of demographic differences should be cautiously drawn, due to the risk of lack of power reducing the chances of detecting a true effect [30]. Thereto, included cases can be seen as a selected, more healthy population than general CVD cases, as they survived the event. Finally, a potential limitation is the use of replacement of controls (hence a control can be used as control several times), contributing to an increased risk of lower precision. However, using controls several times can contribute to stronger validity [31].

## Conclusion

Individuals who experienced a CVD event had a higher prevalence of unhealthy lifestyle habits and other lifestyle-related factors prior to the CVD event, compared to matched controls. However, cases improved their lifestyles to a greater extent compared to their controls, indicating that a CVD event may provide an opportunity for individuals to change lifestyle habits. Nonetheless, the prevalence of unhealthy lifestyle habits was still high in both cases and controls, which emphasizes the need to improve implementation of CVD prevention interventions.

## Supplementary Information

**Additional file 1. **

**Additional file 2. **

## Data Availability

The datasets generated during and/or analysed during the current study are not publicly available as they are the property of the HPI Health Profile Institute, but are available from the corresponding author, amanda.lonn@gih.se, on reasonable request.

## Abbreviations

BMI: Body mass index
CVD: Cardiovascular disease
HPA: Health profile assessment
