# Supplementary material for: Lifestyle-related habits and factors before and after cardiovascular diagnosis: a case control study among 2,548 Swedish individuals
Source: Int J Behav Nutr Phys Act. 2023 Apr 5;20:41. doi: 10.1186/s12966-023-01446-w (PMC10074688; doi:10.1186/s12966-023-01446-w)
Supplement: Supplementary file 1 — Additional file 1. [file 12966_2023_1446_MOESM1_ESM.docx]

***Additional file 1***

***Questionnaire:***

**I exercise for the purpose of maintaining/improving my physical fitness, health and well-being**

Never; Sometimes; 1–2 times/week; 3–5 times/week; At least 6 times/week

**I walk or cycle to and/or from work:**

Less than 5 minutes per day; 5-9 min/day; 10-19 min/day; 20-29 min/day; at least 30 min/day

**I smoke:**

At least 20 cig/day; 11–19 cig/day; 1–10 cig/day; Occasionally; Never

**I consider my diet (regarding both meal frequency and nutritional content) to be:**

Very poor; Poor; Neither good nor bad; Good; Very good

**I consider my alcohol habits to be:**

Very poor; Poor; Neither good nor bad; Good; Very good

**I perceive my physical and mental health as:**

Very poor; Poor; Neither good nor bad; Good; Very good

**I experience stress in my life in total, work included:**

Very often; Often; Now and then; Rarely; Never
